# Supplementary material for: Porous Organosilica Films: Is It Possible to Enhance Hydrophobicity While Maintaining Elastic Stiffness?
Source: Polymers (Basel). 2025 Sep 8;17(17):2433. doi: 10.3390/polym17172433 (PMC12431103; doi:10.3390/polym17172433)
Supplement: Supplementary file 1 [file polymers-17-02433-s001.zip › polymers-3837909-supplementary.pdf]

polymers

MDPI

Supplementary Materials

Porous Organosilica Films: Is It Possible to Enhance Hydrophobicity While Maintaining Elastic Stiffness?

Alexey S. Vishnevskiy *, Dmitry A. Vorotyntsev, Dmitry S. Seregin, Konstantin A. Vorotilov and Alexander S. Sigov

Research and Educational Center "Technological Center", MIREA—Russian Technological University (RTU MIREA), 78 Vernadsky ave., Moscow 119454, Russia; vorotyncev@mirea.ru (D.A.V.); d_seregin@mirea.ru (D.S.S.); vorotilov@mirea.ru (K.A.V.); assigov@yandex.ru (A.S.S.)
* Correspondence: vishnevskiy@mirea.ru

S1. Detailed Description of the Film-Forming Solution Preparation Procedure

Film samples were prepared from film-forming solutions using chemical solution deposition (CSD) techniques. Two copolymers were selected for the experiments. The first copolymer was synthesized from tetraethoxysilane (TEOS, 99.999%, Sigma-Aldrich, St. Louis, MO, USA) and methyltriethoxysilane (MTEOS, 99%, Sigma-Aldrich, St. Louis, MO, USA) via sol–gel reactions. Hydrolysis and copolymerization of TEOS and MTEOS result in a silicon oxide network with terminal methyl groups, as the Si–CH_3 bond is not hydrolyzed. The second copolymer was also synthesized based on TEOS, but instead of MTEOS, a precursor with two terminal methyl groups, namely diethoxydimethylsilane (DEDMS, 97%, Acros Organics, Geel, Belgium), was used. Accordingly, this copolymer contains dimethyl rather than monomethyl groups.

To prepare the colloidal TEOS/MTEOS (series 'm') and TEOS/DEDMS (series 'd') sols, H_2O (Chromasolv® Plus, for HPLC, Sigma-Aldrich, St. Louis, MO, USA) and HCl (37 wt% aqueous solution, Primar Plus™, Trace Analysis Grade, Fisher Scientific, Loughborough, UK) were first dissolved in isopropanol (C_3H_7OH, semiconductor-grade VLSI Puranal, Sigma-Aldrich, St. Louis, MO, USA) using a magnetic stirrer. Next, TEOS and MTEOS (for the 'm' solution) were added dropwise as pure, undiluted liquids to the acidified water-isopropanol mixture under vigorous stirring at room temperature, while TEOS and DEDMS were slowly added in the case of the 'd' solution. The sols were then placed on a magnetic stirrer heated to 60 °C and continuously stirred at medium speed for 3 hours in a closed vessel. Afterwards, the sols were allowed to cool naturally to room temperature. Once cooled, the sacrificial surfactant Brij® L4 ($C_{12}H_{25}(OCH_2CH_2)_4OH$, molecular weight 362, Sigma-Aldrich, St. Louis, MO, USA) was added at 19 wt% = 100 × m_{Brij} / (m_{Brij} + m_{TEOS} + $m_{MTEOS\ or\ DEDMS}$) to induce porosity in these copolymer films via evaporation-induced self-assembly (EISA) [32]. Finally, the solutions were diluted by adding isopropanol to achieve silicon concentrations (Si%) of approximately 6.5 and 1.2 wt% for the relatively thick and thin films, respectively. The silicon concentration was calculated as Si% = 100 × 28 × ($n_{MTEOS\ or\ DEDMS}$ + n_{TEOS}) / m, where m refers to the mass of the 'm' or 'd' solution. All precursors were used as received, without any further purification.

The ratio CH_3/Si = n_{MTEOS} / (n_{MTEOS} + n_{TEOS}) = 2 × n_{DEDMS} / (n_{DEDMS} + n_{TEOS}) was 0.2, 0.6, and 1.0 for both solutions. Here, n_{MTEOS} represents the moles of MTEOS, n_{TEOS}—moles of TEOS, n_{DEDMS}—moles of DEDMS. The molar ratios were [TEOS + MTEOS]:H_2O:HCl = [TEOS + DEDMS]:H_2O:HCl = 1:4:0.002. The amounts of rea-

Academic Editor: Hyeon Mo Cho

Received: 8 August 2025
Revised: 2 September 2025
Accepted: 4 September 2025
Published: 8 September 2025

Citation: Vishnevskiy, A.S.; Vorotyntsev, D.A.; Seregin, D.S.; Vorotilov, K.A.; Sigov, A.S. Porous Organosilica Films: Is It Possible to Enhance Hydrophobicity While Maintaining Elastic Stiffness? *Polymers* **2025**, *17*, 2433. https://doi.org/10.3390/polym17172433

Copyright: © 2025 by the authors. Licensee MDPI, Basel, Switzerland. This article is an open access article distributed under the terms and conditions of the Creative Commons Attribution (CC BY) license (https://creativecommons.org/licenses/by/4.0/).

gents used to prepare ~500 mL of film-forming solutions containing ~6.5 wt% Si are presented in Table S1.

Table S1. Amounts of reagents used to prepare ~500 mL of film-forming solutions containing ~6.5 wt% Si.

Sol series	CH_3/Si ratio	TEOS	MTEOS	DEDMS	C_3H_7OH	H_2O	HCl	Brij® L4 (g)
		(mL)						
	0.2	161	36	-	237	72	0.056	42.3
‘m’	0.6	81	109	-	243	66	0.056	41.6
	1.0	-	185	-	256	59	0.057	44.9
	0.2	189	-	16.2	216	76	0.054	44.0
‘d’	0.6	163	-	54.4	207	75	0.060	45.5
	1.0	115	-	88.5	231	65	0.058	43.4

In this table: TEOS—tetraethoxysilane, MTEOS—methyltriethoxysilane, DEDMS—diethoxydimethylsilane.

Disclaimer/Publisher’s Note: The statements, opinions and data contained in all publications are solely those of the individual author(s) and contributor(s) and not of MDPI and/or the editor(s). MDPI and/or the editor(s) disclaim responsibility for any injury to people or property resulting from any ideas, methods, instructions or products referred to in the content.
